# Administration of eptifibatide as rescue therapy during mechanical thrombectomy in acute ischemic stroke

**DOI:** 10.3389/fneur.2025.1671111

**Published:** 2025-11-26

**Authors:** David Cernik, Jiri Neumann, Jarmila Neradova, Veronika Hlinena, Pavol Viglas, David Cihlar, Filip Cihlar

**Affiliations:** 1 Department of Neurology, Univerzita Palackeho v Olomouci Lekarska fakulta, Olomouc, Czechia; 2 Department of Neurology, Masarykova nemocnice Usti nad Labem oz, Ústí nad Labem, Czechia; 3 Department of Neurology, Krajska zdravotni as Nemocnice Chomutov oz, Chomutov, Czechia; 4 Department of Physical Education and Sport, Faculty of Education, Univerzita Jana Evangelisty Purkyne v Usti nad Labem, Ústí nad Labem, Czechia; 5 Department of Radiology, Faculty of Medicine in Hradec Kralove, Charles University, Hradec Kralove, Czechia; 6 Department of Radiology, J. E. Purkinje University, Masaryk Hospital Krajska zdravotni as, Ústí nad Labem, Czechia

**Keywords:** eptifibatide, mechanical thrombectomy, endovascular treatment, stroke, glycoprotein IIb/IIIa inhibitors

## Abstract

**Background:**

The success of the technical implementation of mechanical thrombectomy (MT) depends on many factors. Considering the experience of the interventional radiologist and the anatomical vascular differences of individual patients, it has technical limits. In our group, we focused on verifying the effectiveness and safety of eptifibatide intra-arterial administration when a sufficient degree of recanalization was not achieved with standard instruments.

**Methods:**

The study included 1,350 patients who underwent MT between 2008 and 2022. Neurological deficit was assessed with National Institutes of Health Stroke Scale (NIHSS) and clinical outcome with modified Rankin scale (mRS). Presence of symptomatic intracerebral hemorrhage (SICH) was assessed according to the SITS-MOST criteria. In some patients, eptifibatide was administered in the case of insufficient recanalization or in the case of an acute periprocedural tendency to reocclusion. Achieved recanalization using the Thrombolysis in Cerebral Infarction scale (TICI).

**Results:**

Eptifibatide was administered during the procedure to 181 patients (age 67.8 ± 12.2 years, 57.5% men). In the eptifibatide group, successful recanalization (≥TICI2b) was achieved in 80.1%, the incidence of ICH was 21.6% and SICH 3.9%. In the group without eptifibatide, successful recanalization was achieved in 80.5%, ICH 19.9% and SICH 5.2%.

**Conclusion:**

The use of eptifibatide is an effective and safe procedure in technically more difficult cases of MT. It enables a similar degree of recanalization to be achieved without increasing the risk of SICH.

## Introduction

Mechanical thrombectomy (MT) is a standard, highly effective and safe treatment for symptomatic cerebral artery occlusion in patients with acute ischemic stroke (AIS). Unfortunately, not all patients achieve a good clinical outcome. One of the most important factors is successful recanalization. Currently, a wide range of instruments are available, which differ in size, design and principle of use. The choice of instrument or technique can have a major impact on the outcome of the intervention. In positive randomized studies, stent-retrievers were used exclusively, which are currently completely dominant worldwide. Another technique is aspiration thrombectomy. In 2017, a randomized study ASTER (The Contact Aspiration vs. Stent Retriever for Successful Revascularization) was published, which did not demonstrate a significant difference in the number of achieved recanalizations between stent-retrievers and aspiration, however, patients treated with aspiration more often needed additional treatment compared to stent-retrievers (1). The combination of aspiration and stent-retriever after failure of the primary thrombectomy method can increase the success rate of recanalization, so some centers primarily use various combinations of aspiration thrombectomy and stent-retriever, e.g., the ARTS (Aspiration-Retriever Technique for Stroke, ARTS) method (2). Despite the use of various techniques, a significant proportion of procedures still fail to achieve successful recanalization of the occluded artery. Currently, the ADAPT (A Direct Aspiration first-Pass Technique) technique is increasingly used, where the primary recanalization technique is the use of aspiration (6). Despite various techniques and instruments, a significant proportion of patients still fail to achieve a sufficient level of recanalization. Eptifibatide is a glycoprotein IIB/IIIA receptor antagonist that is used in combination with a fibrinolytic agent to treat myocardial infarction. Efforts to improve recanalization rates by administering eptifibatide (or other glycoprotein IIb/IIIa inhibitors) in endovascular procedures have generally not yielded conclusive results. The approach in our work is different. Eptifibatide is administered only in the case of primary endovascular failure. The aim of the work is to show that this rescue technique can achieve a significant degree of recanalization in previously almost hopeless cases.

## Methods

Patients with acute ischemic stroke due to large vessel occlusion who underwent mechanical thrombectomy were included into a monocentric retrospective study. Intravenous thrombolysis was administered to most patients.

Initial neurological deficit was assessed using the National Institutes of Health Stroke Scale (NIHSS). The achieved recanalization was evaluated using the Thrombolysis in Cerebral Infarction scale (TICI). Eptifibatide (Integrilin, Glaxo Operations UK Ltd.) was applied as rescue therapy after careful consideration of the risks in case of unsuccessful recanalization (TICI <2b) or a clear tendency for immediate reocclusion already during the endovascular procedure.

The indication criteria for the use of eptifibatide were intracranial artery occlusion intractable to mechanical thrombectomy, periprocedural rethrombosis of atherosclerotic stenosis of intracranial arteries, implantation of an intracranial stent with a clear periprocedural tendency to reocclusion, acute stent thrombosis of carotid or vertebral arteries. In the case of rethrombosis of stenosis and occlusion of the intracranial artery, eptifibatide was administered intraarterially according to the effect of the treatment. The maximum dose for an i.v. bolus (180 micrograms/kg) was not exceeded. Continuous administration was followed by continuous infusion at a dose of 2 micrograms/kg/min if the bolus intra-arterial administration was ineffective until the patient was switched to dual antiplatelet therapy (but not longer than 8 h). Before starting eptifibatide administration, cone beam CT was performed since 2015 to exclude periprocedural hemorrhage.

We evaluated the safety of the chosen procedure (occurrence of bleeding), the resulting degree of recanalization at the end of the procedure and the clinical effect.

Symptomatic intracerebral hemorrhage (SICH) on follow-up CT scan 24 h later was assessed according to the SITS-MOST criteria. The presence of intracerebral hemorrhage (ICH) was strictly defined as any new hyperdensity on follow-up CT. As part of secondary prevention, a statin and antiplatelet therapy were used (preferably dual antiplatelet therapy in the form of acetylsalicylic acid and clopidogrel, if ICH was not present on the control CT). In the case of ICH, antiplatelet monotherapy (acetylsalicylic acid) was used at a safe interval according to the size of the ICH. In the case of cardioembolization etiology of AIS, secondary anticoagulant prevention was preferred. Clinical outcome was assessed using the modified Rankin Scale (mRS).

The aim of the study was to determine efficacy, qualified by achieving successful recanalization of TICI2b ≥2b and achieving self-sufficiency at a three-month interval, assessed by the mRS scale ≤2. The second, equally important endpoint was the assessment of safety, qualified by the rate of hemorrhagic complications during rescue therapy.

STATISTICA 13.0 (Tibco Software Inc., 2018, United States) was used for statistical data processing. The Shapiro–Wilk test was used to determine the normality of the data. Due to the non-normal distribution of frequencies, non-parametric statistical procedures were used. The Mann Whitney test was used to determine any differences and the chi-square test for possible connections. For both tests, statistical significance was defined as *p* < 0.05.

## Results

Patients who underwent mechanical thrombectomy from 2008 to 2022 were included in the study.

Of the 1,350 MTs, 409 patients (30.3% of all MTs performed) failed to achieve successful recanalization. 181 patients were given eptifibatide (44.3% of MTs with primary inadequate recanalization) and 145 of them subsequently achieved successful recanalization (35.5% of MTs with primary inadequate recanalization, 80.1% of MTs in which eptifibatide was administered).

Patients with eptifibatide were younger (67.8 vs. 73.7%, *p* < 0.00001), more often male (57.5 vs. 45%, *p* = 0.002), with a milder baseline clinical condition (15 vs. 17 points in NIHSS, *p* = 0.009). Patients also suffered from fewer comorbidities. The incidence of arterial hypertension was lower (69.6 vs. 77.4%, *p* = 0.021), the incidence of atrial fibrillation was also lower (22.1 vs. 38.9%, *p* = 0.00001) and occlusion in the carotid basin was significantly less common (76.8 vs. 89.8%, *p* < 0.00001). There were also significant differences in previous use of anticoagulant therapy (6.6 vs. 15.7, *p* = 0.001) and administration of intravenous thrombolysis before endovascular procedure (68 vs. 76.9%, *p* = 0.009). The basic and demographic characteristics of the group are summarized in Table 1.

**Table 1:** Demographic and baseline clinical characteristics of enrolled patients.

	With eptifibatide	Without eptifibatide	*p*
Number	181	1,169	
Age (median ± SD)	67.8 ± 12.2	72.1 ± 12.4	<0.00001
Male (%)	57.5%	45%	0.0017
Diabetes mellitus (%)	26.5%	30.1%	0.324
Arterial hypertension (%)	69.6%	77.4%	0.021
Ischemic heart disease (%)	24.9%	31.4%	0.076
Hyperlipidemia (%)	63.5%	60%	0.361
Atrial fibrillation (%)	22.1%	38.9%	0.00001
Antiaggregation (%)	26%	30.4%	0.228
Anticoagulation (%)	6.6%	15.7%	0.001
IV thrombolysis (%)	68%	76.9%	0.009
Admission NIHSS (median)	15	17	0.009
Carotid circulation (%)	76.8%	89.8%	<0.00001

SD, standard deviation; IV, intravenous; NIHSS, National Institute of Health Stroke Scale.

In the group initially treated with endovascular failure, successful recanalization was achieved in 80.1% (TICI ≥2b) thanks to the administration of eptifibatide. No statistically significant difference was found compared to the group without eptifibatide (80.5%, *p* = 0.903).

The incidence of complicating intracerebral hemorrhage was statistically insignificantly higher in the eptifibatide group (21.6 vs. 19.9%, *p* = 0.614). However, the incidence of symptomatic intracerebral hemorrhage was statistically insignificantly lower (3.9 vs. 5.2%, *p* = 0.439).

The clinical outcome tended to be better in the eptifibatide group, but no statistically significant difference was found. Good clinical status was achieved by 49.2 vs. 44.7% (mRS ≤2, *p* = 0.256) at 3 months. Relatively good outcome (mRS ≤3) was achieved by 58.6 vs. 50.9%, when the difference was already on the verge of statistical significance (*p* = 0.055). Three-month mortality was also lower (28.7 vs. 33.7%, *p* = 0.185). The results are summarized in Table 2.

**Table 2:** Results—comparison between the patients with eptifibatide and standard treatment.

	With eptifibatide	Without eptifibatide	*p*
Number	181	1,169	
TICI ≥2B (%)	80.1%	80.5%	0.903
ICH (%)	21.6%	19.9%	0.614
SICH (%)	3.9%	5.2%	0.439
3-month mRS ≤2 (%)	49.2%	44.7%	0.256
3-month mRS ≤3 (%)	58.6%	50.9%	0.055
3-month mRS median	3	3	0.364
3-month mortality (%)	28.7%	33.7%	0.185

TICI, Thrombolysis in Cerebral Infarction; mRS, modified Rankin Scale; NIHSS, National Institute of Health Stroke Scale; ICH, intracerebral hemorrhage; SICH, symptomatic intracerebral hemorrhage.

## Discussion

In our study, no difference in the recanalization rate was found between the eptifibatide group and the group without eptifibatide. However, this result is excellent with regard to our basic criterion for administering eptifibatide (primary failure of the basic endovascular procedure). Without salvage therapy, successful recanalization would not have occurred in this group. With the use of eptifibatide, the rate of successful recanalization reached 80%. The clinical outcome is significantly dependent on the success of recanalization. Full self-sufficiency was achieved with the use of salvage therapy in 49% of patients (without successful recanalization, the chance of a good clinical outcome is at the level of a few percent according to common clinical experience). The safety profile of the selected treatment was favorable. There was no significantly higher incidence of ICH and SICH. The number of ICHs was numerically slightly higher in the eptifibatide group, however, the difference was not statistically significant. Such a small difference may also be significantly influenced by the asymmetry of the two groups (there are numerically one order of magnitude fewer patients with eptifibatide).

Ma et al. (3) in a matched control analysis show the effectiveness of eptifibatide administration within the framework of endovascular procedure (162 patients, 81 with eptifibatide and 81 controls, successful recanalization 91.3% versus 81.5%). No significant difference in ICH or SICH was found. Latacz et al. (4) in a cohort with AIS treatment for tandem lesions (115 patients with eptifibatide) also did not find an increased incidence of ICH or SICH. Rana et al. (5) (54 patients with eptifibatide for MT) also report a favorable safety profile with the use of eptifibatide (no significant difference in the incidence of ICH or SICH compared to the two control groups). The mentioned studies are consistent with our safety results. They also show efficacy similar to our case. However, there is a fundamental difference in the approach to administering eptifibatide. In our case, it is only in the case of primary failure of mechanical thrombectomy. For the same reason, we cannot compare the clinical results with these studies. Here again, we can state that the patients who were administered eptifibatide largely benefited.

In our study, patients with eptifibatide tended to have a better clinical outcome (58.6 vs. 50.9%, assessed for mRS ≤3, *p* = 0.055). However, this group was younger and had fewer significant comorbidities (see Table 1).

The limitations of our study are primarily its retrospective nature and the absence of an appropriate control group. The administration of eptifibatide was entirely at the discretion of the interventional radiologist. As shown by the basic characteristics of the group, there is a certain tendency to treat younger patients more intensively. Another limitation of the study is that clinical status was monitored only after 3 months. However, clinical outcome is traditionally dependent on the achievement of recanalization, not on the method by which it is achieved. Due to the safety profile of the treatment, longer follow-up is irrelevant. Given the indication for eptifibatide administration only as salvage therapy, based on our data we cannot clearly comment on the general benefit of eptifibatide as adjuvant therapy to MT, nor was this our goal. However, our data show that if eptifibatide is rationally administered, it does not carry a statistically significant risk of hemorrhagic complications and, on the contrary, provides a very significant chance of successful recanalization.

## Conclusion

Despite advances in endovascular treatment, there are still patients in whom this procedure fails. In our study, eptifibatide was used as salvage therapy in primary failure of endovascular treatment. In 80% of patients primarily without the possibility of recanalization with standard endovascular techniques, this procedure led to successful recanalization. Given the above limitations, we draw two conclusions from our data. The administration of eptifibatide, after careful individual consideration, can be a highly effective salvage therapy in primary failure of mechanical thrombectomy (the last therapeutic option). The second conclusion is the favorable safety profile of the treatment in this indication, which is consistent with the general results of previously published studies using eptifibatide as adjuvant therapy to mechanical thrombectomy. A larger randomized trial would be needed to verify the results.

## Data Availability

The raw data supporting the conclusions of this article will be made available by the authors, without undue reservation.
